# Dasatinib-induced nephrotic syndrome in a patient with chronic myelogenous leukemia: a case report

**DOI:** 10.1186/s12882-019-1273-6

**Published:** 2019-03-07

**Authors:** Shoko Ochiai, Yuji Sato, Akihiro Minakawa, Akihiro Fukuda, Shouichi Fujimoto

**Affiliations:** 1 Department of Internal Medicine, Division of Circulatory and Body Fluid Regulation, Faculty of Medicine, University of Miyazaki, 5200 Kihara, Kiyotake, Miyazaki, 889-1692 Japan; 2 Dialysis Division, University of Miyazaki Hospital, Miyazaki, Japan; 3 Department of Hemovascular Medicine and Artificial Organs, Faculty of Medicine, University of Miyazaki, Miyazaki, Japan

**Keywords:** Drug-related nephrotic syndrome, Tyrosine kinase inhibitor, Dasatinib, Fibrillary glomerulonephritis, Nephrotic syndrome, Vascular endothelial growth factor

## Abstract

**Background:**

Dasatinib is a second-generation tyrosine kinase inhibitor that is indicated for the treatment of patients with chronic myeloid leukemia. Here, we report the case of a man with nephrotic syndrome that was caused by dasatinib.

**Case presentation:**

A 40-year-old man with chronic myeloid leukemia was referred to our hospital because of proteinuria 1 month after dasatinib therapy was introduced. A percutaneous kidney biopsy was performed, diffuse glomerular endothelial injury and effacement of the foot process were noted, and the patient was diagnosed with dasatinib-induced nephrotic syndrome. Additionally, in an electron microscopy study, randomly arranged fibrils were observed in the mesangial and subendothelial regions. Switching from dasatinib to nilotinib led to a decrease in the proteinuria level, from 12 to 0.6 g/g creatinine, within 2 weeks. The patient was discharged from our department on the 25th day after hospitalization, without any drug aftereffects.

**Conclusions:**

Drug-related nephrotic syndrome should be considered when nephrotic syndrome develops during treatment with dasatinib.

## Background

Chronic myelogenous leukemia (CML) is an important hematological tumor that is characterized by the BCR-ABL chimera gene and occurs in 15 to 20% of adults with leukemia [1]. CML slowly progresses in the first 4–6 years after onset, but when it progresses to the blastic stage, it is acute and lethal. The BCR-ABL gene is formed by mutual translocation between c-ABL on chromosome 9 and BCR on chromosome 22, which activates tyrosine kinase continuously, leading to leukemia-cell proliferation [2]. Therefore, tyrosine kinase inhibitors (TKIs) are known to be“silver bullet” therapies that dramatically improve the prognosis of patients with CML. Dasatinib, a second-generation TKI, is available in Japan and leads to a higher remission rate of CML than former-generation TKIs do [3]. However, recent reports suggested that renal adverse effects, including nephrotic syndrome, could occur after TKIs are used [4–10]. Here, we report the case of a CML patient in whom nephrotic syndrome was induced by dasatinib, along with a short literature review.

## Case presentation

A 40-year-old Japanese man was admitted to another hospital with edema of both lower extremities, a feeling of abdominal fullness, and shortness of breath upon exertion. A physical examination showed a giant splenomegaly that reached the pelvic cavity and hepatomegaly. The patient’s leg edema was thought to be caused by the giant splenomegaly. A blood test showed an elevated white blood cell count of 480,000/μL. According to a bone marrow examination, he was diagnosed with CML, and dasatinib at a daily dose of 100 mg was prescribed.

CML was improved with the use of dasatinib therapy, with a decreased volume of splenomegaly and leg edema. However, 1 month after dasatinib was introduced, heavy proteinuria (urinary protein-creatinine ratio of 8.93 g/g creatinine [g/gCr]) appeared, accompanied by a low serum albumin level of 2.3 g/dL. Two months after dasatinib was introduced, the drug was transiently stopped because of pancytopenia, and then the drug was restarted. The patient was referred to our hospital for a further investigation for nephrotic syndrome after dasatinib was used for 3 months.

**Fig. 1:**
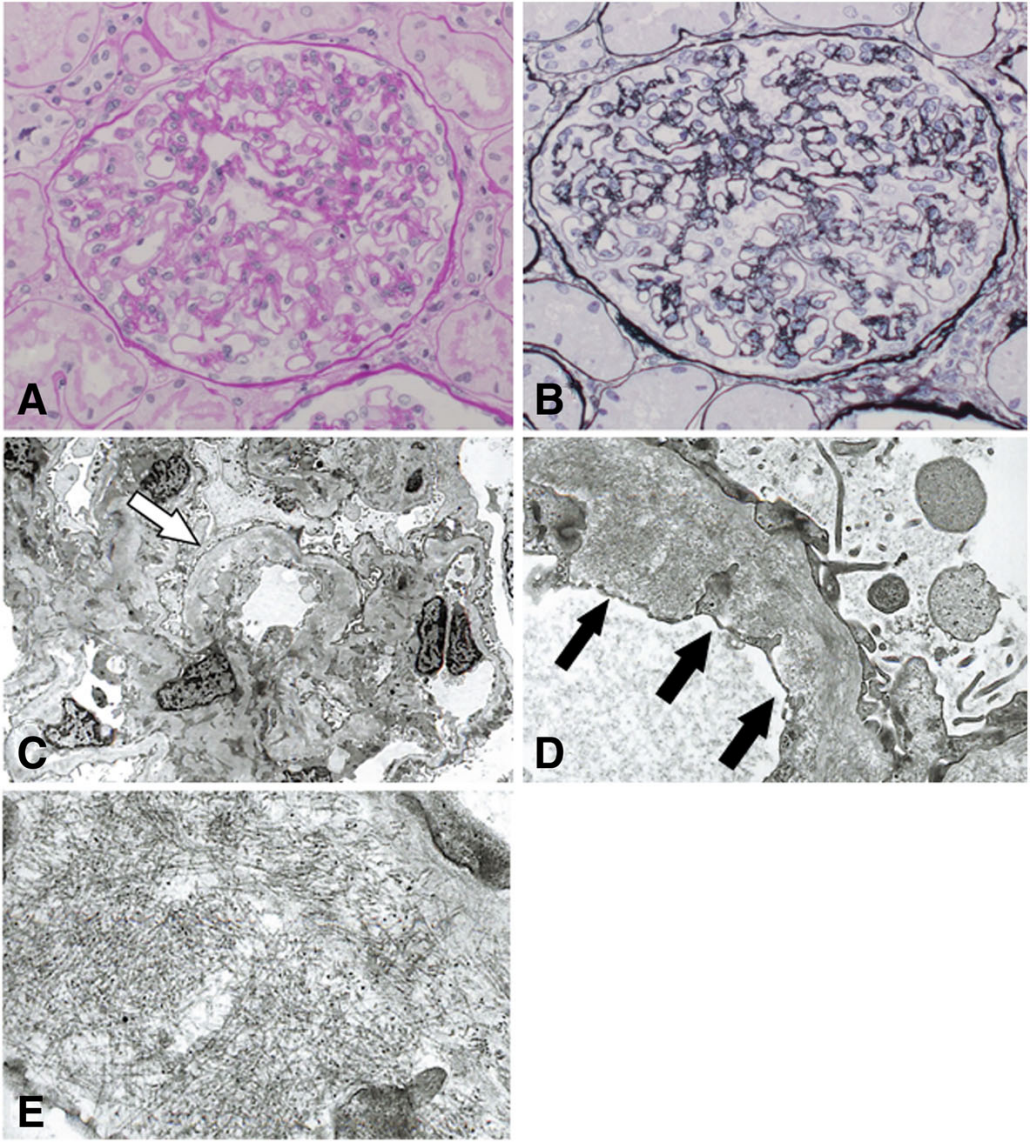
Light microscopic findings with periodic acid–Schiff staining (**a**) revealed diffuse and global endothelial cytoplasm expansion accompanied by focal duplication of the glomerular basement membrane. Spike formation was not evident on the periodic acid-methenamine-silver stained section (**b**). Electron microscopy revealed swelling of the endothelial cells (**c**, arrow) and effacement of the podocyte foot process (**d**). In addition, randomly arranged fibrils (10–20 nm, arrows) are observed in the mesangial, subepithelial and subendothelial regions (**d** and **e**). Original magnification **a**:× 400, **b**:× 400, **c**:× 1000, **d**:× 5000, **e**:× 15000

**Fig. 2:**
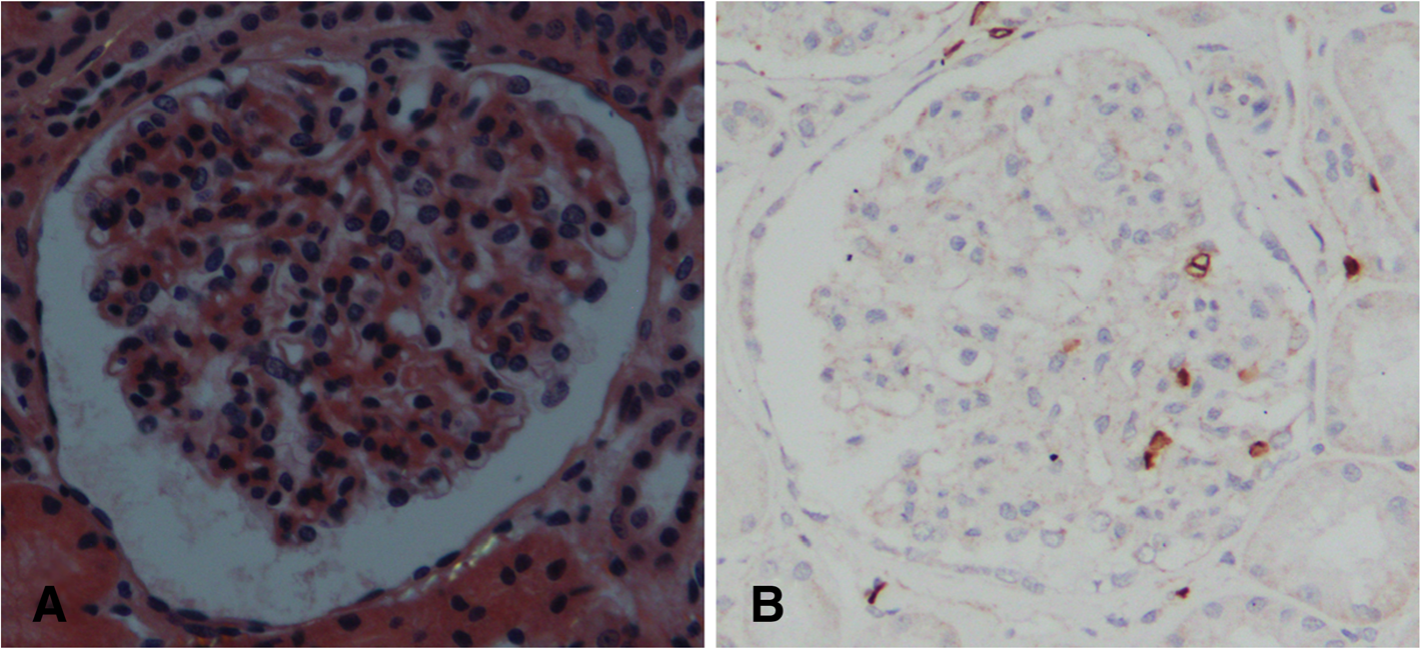
Our case does not show glomerular staining for Congo red (**a**) and DNAJB9 (**b**) immunohistochemistry. (**a**, **b**, Original magnification × 400)

On admission, a physical examination showed a blood pressure of 121/70 mmHg, pulse of 56/min, temperature of 36.5°C, and respiratory rate of 16/min, as well as slight splenomegaly and lower leg edema. A complete blood count test showed a white blood cell count of 11,500/μL, hemoglobin level of 13.2 g/dL, and platelet count of 132,000/μL. Blood chemistry and serology tests showed a serum creatinine level of 0.87 mg/dL (estimated glomerular filtration rate of 78.4 mL/min/1. 73m^2^), serum albumin level of 3.08 g/dL, total cholesterol level of 287 mg/dL, and low-density lipoprotein cholesterol level of 166 mg/dL. In addition, antinuclear antibody and viral hepatitis were not evident in a serological test. A spot urine examination showed protein/creatinine ratio of 12.2 g/gCr, urinary protein 3+, and hematuria 1+. Urine sediment showed 5–9 red blood cells/high-power field and hyaline casts. Ultrasonography showed that the kidneys were almost normal-sized. A percutaneous kidney biopsy was performed, and light microscopy with periodic acid-Schiff staining revealed diffuse and global endothelial cytoplasm expansion that was accompanied by focal duplication of the glomerular basement membrane (Fig. 1a). However, spike formation was not evident in the periodic acid-methenamine silver-stained section test (Fig. 1b). An immunofluorescence study did not show deposition of immunoglobulin or its complement. Electron microscopy revealed swelling of the endothelial cells (Fig. 1c) and effacement of the podocyte foot process (Fig. 1d). In addition, randomly arranged fibrils (10–20 nm) were observed in the mesangial, subepithelial, and subendothelial regions (Fig. 1d and e), but their distribution was relatively focal. AA amyloidosis was deemed unlikely because of negative Congo-red staining and immunohistochemistry (Fig. 2a). In addition, negative immunohistochemistry for DNAJB9 did not suggest the diagnosis of fibrillary glomerulonephritis (Fig. 2b). We thought that these renal histological changes were caused by dasatinib, but the cause and diagnosis of fibril were not evident.

We switched from dasatinib therapy to nilotinib at a daily dose of 600 mg. The patient’s spot urine protein/creatinine ratio improved to 0.63 g/gCr and his hematuria disappeared within 2 weeks. His renal function did not worsen. We concluded that the main cause of nephrotic syndrome was dasatinib because only switch of the drug improved the patient’s proteinuria. He was discharged from our department on the 25th day after hospitalization, without any aftereffects.

## Discussion and conclusions

We reported the case of a patient with nephrotic syndrome that was possibly induced by dasatinib treatment for CML. Dasatinib-related proteinuria was reported in 18% of participants in a phase I dose-escalation and pharmacokinetic study to treat patients with advanced solid tumors [11]. Five case reports of nephrotic syndrome that was caused by dasatinib are available, three of which are on adults and the others are on children [4–8] (Table 1). In all cases, discontinuation or reduction of the dose of dasatinib or a switch to a first-generation TKI improved proteinuria. Hirano et al. proposed that the severity of proteinuria was dose-dependent, because reducing the dose by half was efficient in their study [5]. A kidney biopsy was performed on three of their study subjects, and the study revealed that both endothelial cell injury and effacement of the foot process were common findings, while the severity of effacement of the foot process differed among the cases.

**Table 1:** Clinical features of patients with dasatinib-induced nephrotic syndrome in previous case reports

Case	Author	Patient	Basal hematological disease	Duration of dasatinib administration	Urinary protein excretion	Serum creatinine (mg/dL)	Renal histology	Treatment	Prognosis
1	Wallace^[4]^	63, F	CML	3 months	3.9 g/day	0.79	Focal foot process effacement	Switch to imatinib	remission
2	Hirano^[5]^	64, F	Ph + ALL	2 weeks	3.9 g/day	0.34	NA	Dose reduction	remission
3	Ruebner^[6]^	3, F	CML	17 months	UP/Ucr = 17 g/gCr	0.3	Focal foot process effacement	Discontinue	remission
4	Lim YT^[7]^	5, M	Ph + ALL		UP/Ucr = 15.24 g/gCr	NA	Diffuse foot process effacement	Discontinue	remission
5	De Luca^[8]^	45, F	CML	6 months	4.0 g/day	0.9	NA	Switch to imatinib	remission
6	Our case	40, M	CML	3 months	5.7 g/day	0.87	Endothelial cell injury and foot process effacement	Switch to nilotinib	remission

Abbreviations: *CML* chronic myeloid leukemia, *Ph + ALL* Philadelphia-positive acute lymphoblastic leukemia, UP/UCr: urinary protein: urinary creatinine concentration ratio

Dasatinib is a second-generation TKI, as well as a multi-kinase inhibitor, that inhibits not only the BCR-ABL gene but also other kinases such as the platelet-derived growth factor receptor beta, KIT, and SRC kinase family [12]. Dasatinib inhibits the SRC family of kinases (SFK) as well as the production of vascular endothelial growth factor (VEGF) indirectly through SFK [13]. VEGF is produced in podocytes, binds to the VEGF-2 receptor of endothelial cells with a paracrine effect, and maintains the cellular function and morphology [14]. In addition, as an autocrine effect, VEGF binds to the VEGF-2 receptor and sFlt-1 of its own podocyte, thereby controlling the cytoskeleton and slit diaphragm between the podocyte foot processes [14]. This is the most likely reason why VEGF inhibition by dasatinib causes podocyte and endothelial cell disorders that lead to nephrotic syndrome. Pfister et al. reported the histological characteristics induced by anti-VEGF therapy [15]. They described glomerular capillary microaneurysms and segmental semilunar hyalinoses were most frequently found in anti-VEGF therapy-induced glomerulopathy, but we could not see these changes in our case. However, some of the histological findings, such as endothelial cytoplasm expansion and double contours of glomerular basement membrane, were compatible with those reported by Pfister et al. Even for drugs with the same VEGF inhibition, the renal pathological changes caused by different drugs might not be the same. In our case, endothelial cell and podocyte injury might be reversible because proteinuria is decreased by discontinuing the drug or reducing the dose. First-generation TKIs are not capable of inhibiting VEGF [16, 17]; therefore, switching to a first-generation TKI is another effective treatment method. Some people who use dasatinib have mild proteinuria and others experience progression to nephrotic syndrome, but the mechanism of various degrees of proteinuria is unknown. However, massive proteinuria is caused by massive podocyte injury, not by endothelial cell injury [18, 19]. The electron microscopy study of our case showed diffuse effacement of the foot process. We think this effacement caused massive proteinuria. Because endothelial cells could be more easily injured by the small doses of dasatinib than podocytes, short-term administration of dasatinib or a low dose of dasatinib may not cause nephrotic-range proteinuria, but cause only mild proteinuria. Then, an injury that progresses to the podocytes could cause a greater amount of proteinuria.

In addition, we found a glomerular deposit (called fibril) in the EM study, which was negative for a Congo-red staining, suggesting non-amyloid deposit. At first, we suspected the fibril may be consistent with a diagnosis of fibrillary glomerulonephritis (FGN), because the diameter of the fibril seen in this case was larger than that seen in amyloidosis (φ10–20 nm vs φ8–12 nm). FGN is observed in 0.6–1.0% of kidney biopsies in Europe and the United States [20, 21]. In general, there are reports that patients with FGN have a poor prognosis, and 44% of these patients experience end-stage renal disease [22]. FGN has been reported to be associated with systemic lupus erythematosus, Crohn’s disease, Graves’ disease, or gastric cancer [22]. However, previous reports of dasatinib-induced glomerular damage in CML patients has never reported dasatinib-induced deposits such as FGN [4–8]. Usually, in patients with FGN, the prevalent pathologic finding is mesangial expansion in light microscopy, and the deposition of IgG and/or C3 in immunofluorescence microscopy [21]. However, in our case, those findings were not seen, although there was an endothelial cell injury. In addition, the immunofluorescence microscopy result was negative in our case. DNAJB9 immunohistochemistry, which is specific for FGN [23], was not positive. We found the fibril coincidentally after performing an electron microscopy study. Considering these results, we could not clarify the cause of fibril and the association between the fibril and dasatinib-induced renal damage. We deem that fibrils did not contribute much to proteinuria because the switch to a TKI dramatically decreased the amount of proteinuria. We believe that this case is worthy to report not only because of dasatinib-caused nephrotic syndrome but also because of the coexistence of fibrils in this case.

In summary, we reported a possible case of dasatinib-induced nephrotic syndrome in a patient with CML. Dasatinib has multi-kinase inhibition activity, unlike other TKIs, and it could injure podocyte and endothelial cells via the inhibition of VEGF. Dasatinib is a useful drug for treating CML, and we expected it to be used more frequently in the future. However, we should keep in mind that the drug’s adverse effects, because they are reversible if early intervention is performed.

## Abbreviations

CML: Chronic myelogenous leukemia
Cr: Creatinine
EM: Electron microscopy
FGN: Fibrillary glomerulonephritis
SFK: SRC family kinase
TKI: Tyrosine kinase inhibitor
VEGF: Vascular endothelial growth factor
